# Ten Cases of Biopsy-Proven Acute Tubulointerstitial Nephritis: Report from a Single Center in a Rural Area from 2008 to 2021

**DOI:** 10.1155/2022/6203803

**Published:** 2022-08-05

**Authors:** Kei Nagai, Tsuyoshi Tsukada, Akiko Sakata, Atsushi Ueda

**Affiliations:** ^1^Department of Nephrology, Hitachi General Hospital, 2-1-1 Jonan-Cho, Hitachi, Ibaraki 317-0077, Japan; ^2^Department of Nephrology, Faculty of Medicine, University of Tsukuba, 1-1-1 Tennodai, Tsukuba, Ibaraki 305-8575, Japan; ^3^Department of Pathology, Hitachi General Hospital, 2-1-1 Jonan-Cho, Hitachi, Ibaraki 317-0077, Japan

## Abstract

Acute tubulointerstitial nephritis (ATIN) can be caused by any number of factors, and it accounts for several percent of renal biopsy cases. In Japan, case reports exist, but there are few single-center series of ATIN cases. *Case 1*. A teenage male patient developed fever and cough on day X-61 and was found to have normal renal function and positive C-reactive protein (CRP) by his primary care physician. On day X-20, he presented with cough and nasal discharge in addition to low-grade fever, and his doctor noted renal dysfunction with serum creatinine of 2.12 mg/dL, negative urine occult blood, and positive urine glucose. Renal biopsy results showed diffuse interstitial nephritis with scarce glomerular involvement. There was no concurrent uveitis. Renal function normalized after 4 months of treatment with moderate-dose prednisolone. *Cases 2–10*. Of the 422 cases for which renal biopsies were performed at our institution from 2008 to 2021, acute tubulointerstitial nephritis was confirmed clinically and pathologically in 9 cases in addition to case 1, accounting for 2.4% of all biopsy cases. In the analysis of the 10 patients, the median age was 40 years old, eGFR at diagnosis was 19.4 (3.2–49.1) mL/min/1.73 m^2^, and 2 of them underwent hemodialysis, but both were weaned from dialysis, and the eGFR after treatment was 53.6 (20.8–110.0) mL/min/1.73 m^2^; all patients showed improvement (*P* < 0.001). Treatment consisted of steroids in 8 patients and no steroids in 2 patients, the latter being treated by discontinuation of the suspect drugs and treatment of infection; 7 of the 10 patients were examined for ocular uveitis, and uveitis was diagnosed in 5 patients. The causes and clinical course of ATIN are diverse, but it is treated according to individual judgment in addition to standard treatment, and it generally has a good renal prognosis.

## 1. Introduction 

Acute tubulointerstitial nephritis (ATIN) accounts for approximately 1–3% of all renal biopsies and 6–27% of renal biopsies during the clinical course of acute kidney injury, depending on the patient population [1–4]. Classically, ATIN has been reported to be secondary to bacterial and viral infections and tuberculosis, but in recent years, antibiotics and anti-inflammatory drugs (non-steroidal anti-inflammatory drugs, NSAIDs), as well as proton pump inhibitors (PPIs), have been implicated. Sarcoidosis, IgG4-related disease, and tubulointerstitial nephritis and uveitis (TINU) syndrome have also been reported as immunological causes [1, 5–7]. Differences in causes are reflected in differences in the clinical course, which vary greatly depending on the subject under investigation, and there are fewer reported cases of ATIN than those of glomerular disease. Therefore, reports from specific spatiotemporal areas are important for an accurate understanding of the clinical epidemiology of ATIN. In this report, a representative case and a series of ATIN cases in a single center in a rural area, which have not been reported extensively in Japan to date, are presented.

## 2. Case Presentation

### 2.1. Case 1

**Figure 1:**
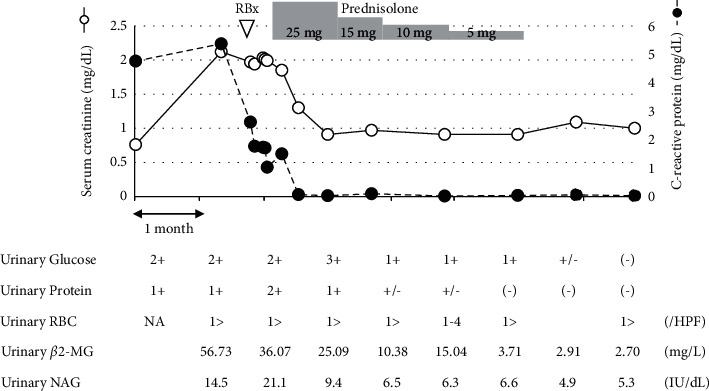
Clinical features and course of case 1. Renal biopsy of case 1 was performed approximately 2 months after disease onset. Disease activity and effectiveness of treatment for acute tubulointerstitial nephritis were monitored by renal function (serum creatinine), inflammation (C-reactive protein), and tubular functional defects (urinary glucose, protein, *β*2-microglobulin, and N-acetylglucosaminidase). Abbreviations: RBx, renal biopsy; RBCs, red blood cells; NA, not available; HPF, high-power field; NAG, N-acetylglucosaminidase.

**Figure 2:**
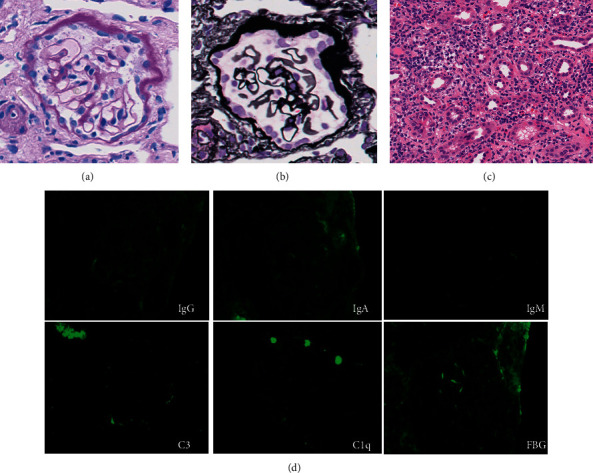
Renal pathological findings of case 1. Light microscopy findings show minor glomerular changes in case 1. Periodic acid-Schiff staining (a, c) and periodic acid methenamine silver staining (b). Immunofluorescence studies show no specific staining for immunoglobulin, complement, and fibrinogen (d).

A teenage male patient developed fever and cough on X-61 and was found to have normal renal function and positive C-reactive protein (CRP) of 4.76 mg/dL by his primary care physician. On day X-20, he presented with cough and nasal discharge in addition to a low-grade fever, and he was found to have serum CRP of 5.37 mg/dL, serum creatinine of 2.12 mg/dL, negative urine occult blood, and positive urine protein (1+) and urine sugar (1+). At the same time, he was on multiple medications, including antitussive expectorants and anti-allergic medications. At the subsequent visit on day X-6, positive CRP and renal impairment continued, and he was referred to our hospital for further investigation. On day X-4, the laboratory data indicated severe tubulointerstitial nephritis with positive urine protein (1+) and urine sugar (1+), along with urine *β*2-microglobulin of 56.73 mg/L and N-acetyl-*β*-D-glucosaminidase (NAG) of 14.5 IU/L, with eosinophilia (11% of white blood cells, 7100/*μ*L) (Figure 1). He was hospitalized for a renal biopsy in our department, and laboratory test findings on admission are shown in Table 1. Anti-nuclear antibody was negative, and specific antibodies (anti-dsDNA-antibody, anti-SS-A, and anti-SS-B antibody) were negative on serum immunology. The renal specimen contained 8 glomeruli and was found to show no global sclerosis and no proliferative lesions or membranous alterations in the glomeruli (Figures 2(a) and 2(b)). The cortical interstitium was moderately diffusely expanded, with inflammatory cell infiltration consisting mainly of lymphocytes with plasma cells, histiocytes, and neutrophils. Diffuse areas of tubulitis were observed, with necrosis and desquamation of tubular epithelial cells (Figure 2(c)). These changes were also seen in the medullary interstitium as tubulitis (not shown). Immunoglobulins and complement were not specifically stained in the glomeruli (Figure 2(d)). Collectively, this was diagnosed as diffuse tubulointerstitial nephritis without glomerular involvement. There was no concurrent uveitis, and other serological findings did not indicate secondary interstitial nephritis such as due to Sjogren's syndrome. Since his renal injury and general symptoms (fever and fatigue) did not remit spontaneously, it was decided to treat him with corticosteroid for his nephritis possibly due to the prior common airway infection and/or a drug-induced mechanism. A low dose of oral prednisolone, 25 mg/day (approximately 0.45 mg/kg/day), was administered on an outpatient basis, and the dose was decreased each month; renal function normalized after 4 months of treatment with the gradually tapered prednisolone. The treatment of case 1 was based on the empirical methods commonly used in our hospital, but with a smaller steroid dose and a longer duration compared to description in textbooks based on case series from other countries [8].

### 2.2. Cases 2–10

Of the 422 cases for which renal biopsies with any reason for examination were performed at our center from 2008 to 2021, ATIN was confirmed clinically and pathologically in 9 cases in addition to case 1, accounting for 2.4% of all biopsy cases (Table 2). In the analysis of 10 patients, the median age was 40 years, eGFR at diagnosis was 19.4 (3.2–49.1) mL/min/1.73 m^2^, and 2 of them underwent hemodialysis, but both patients were withdrawn from dialysis. The median eGFR after treatment was 53.6 (20.8–110.0) mL/min/1.73 m^2^, and all patients showed improvement (*P* < 0.001). Treatment consisted of corticosteroids (15 to 50 mg) in 8 patients; the 2 patients who did not receive corticosteroids were treated by discontinuation of the suspected drugs and treatment of infection. Seven of the 10 patients were evaluated for ocular uveitis, and uveitis was found in 5 of them. Although cases 2 and 6 were elderly patients and the steroid dosage was relatively high compared to the other cases, they relapsed during the outpatient course and required re-treatment.

## 3. Discussion

TIN is a histopathologic disease concept and is a general term for renal lesions that are primarily inflammatory in the tubulointerstitial region. It is classified as ATIN, which mainly consists of acute lesions such as edema and cellular infiltration, and chronic TIN, which consists of chronic changes, such as interstitial fibrosis and tubular atrophy. As in case 1, ATIN can be properly diagnosed by examination of both the clinical and pathological aspects of the case. Causes of ATIN include infections, drug-related, immune abnormalities, urinary tract abnormalities, heavy metal and other poisonings, ischemia, and metabolic disorders [1–4, 8]. Systematic survey reports from western countries have estimated that 71% are antimicrobial and other drug-related, 15% are infectious, 8% are idiopathic, 5% are TINU syndrome, and 1% is sarcoidosis [9]. In Japan, ATIN, which has a wide variety of causes, has no definitive treatment protocols. Corticosteroid therapy has been reported to be effective in ATIN, but there are no controlled trials, and it is difficult to establish the optimal treatment, including its dosage [9]. Steroids are empirically administered at a dose of about 1 mg/kg/day of prednisolone orally for 2 or 3 weeks, followed by a gradually tapering dose over 3 to 4 weeks [10, 11], but small doses or steroid pulse therapy may be administered on a case by case basis.

Case 1 was diagnosed as ATIN due to prior common airway infection and/or drug-induced. In the setting of infection-related interstitial nephritis, eradication of the infection is often associated with renal function recovery. Treatment of drug-induced ATIN is more complicated and controversial. The most important intervention is early recognition of the disease and drug discontinuation. A drug-free trial should be undertaken to determine if renal function recovers without further intervention [8]. If no improvement is noted after a period of observation (several days) or if renal function decreases rapidly, a trial of corticosteroid is reasonable [8]. Despite early intervention including culprit drug discontinuation, a substantial proportion of patients (up to 35%) may develop CKD [8]. In our experience, 5 of 10 cases continued to have decreased renal function, with eGFR less than 60 mL/min/1.73 m^2^ (Table 2). Nevertheless, at our hospital, we do not necessarily use high doses of steroids, but often use 0.4 to 0.6 mg/kg, to induce remission of ATIN and substantial recovery of renal function. Our practical view is to try to eliminate the cause of the disease by taking a detailed history, not to introduce treatment for a week until renal biopsy results are available if renal injury is not severe, and to use outpatient steroid therapy if the exacerbation trend continues.

In addition to renal function as an indicator of treatment, disease-specific clinical findings of ATIN are used to determine the efficacy of interventions. Laboratory findings in ATIN feature eosinophilia, eosinouria on cytopathology, abnormal urinary sediment (hematuria, leukocyturia, and leukocyte casts), and mild proteinuria (less than 1 g/24 h). Tubular functional defects are represented by glycosuria, phosphaturia, hyperkalemia, acidosis, diabetes insipidus, and increased NAG and *β*2-microglobulin levels [8]. In case 1, a proximal tubular defect was observed as glycosuria and elevated NAG and *β*2-microglobulin levels. These abnormalities remitted gradually with corticosteroid therapy, suggesting that there is no guarantee that the amount of steroids is sufficient, but at least the required amount is reached.

A feature of this case series is that the patients were observed at our hospital, the only facility performing renal biopsies in a rural medical area of approximately 250,000 people, and thus they are less subject to bias due to indications for performing renal biopsies and inconsistent referral criteria to nephrologists than in a multicenter study. In the context of ATIN, we do not have any specific criteria for referral to nephrologist so far. However, our medical area (Hitachi, Japan) has recently developed collaboration between generalist and nephrologist to aim for improving prognosis of chronic kidney disease [12]. We believe that this collaboration will be effective in the early detection, diagnosis, and treatment of ATIN as well. The rate of TINU syndrome was much higher than expected based on previous reports from outside Japan (50% vs. 5% [9], 3% [1], and 2% [13], respectively). No papers have reported accurate international comparisons or ethnic differences in TINU, and it is difficult to generalize when differences in indications for renal biopsy and ophthalmological examination are taken into account, but there is no doubt that TINU is often diagnosed, at least in our region. However, familial occurrence or genetic background, that may involve in development of TINU syndrome [14], was not examined in this case series. We were unable to discuss exactly why our cases were older and male-dominant than the other evidence (young and female-dominant) [15]. Convincing clinical evidence for a role for steroid therapy, however, comes from studies of the idiopathic form of ATIN, particularly TINU syndrome. These manifestations of the cause of renal disease suggest the reason for the effectiveness of low-dose steroids in the present case series for improvement of renal function.

In conclusion, the causes and clinical course of ATIN are diverse, but it is treated according to individual judgment in addition to standard treatment and generally has a good renal prognosis. This report presents the results of treatment at a single institution in Japan, and treatment with a small dose of steroids results in recovery of renal function.

## Figures and Tables

**Table 1:** Laboratory findings of case 1.

Urinalysis		Blood chemistry tests (cont.)	
Gravity	1.016	Sodium	138 mmol/L
Protein	2+	Chloride	102 mmol/L
Sugar	2+	Potassium	3.4 mmol/L
Blood	Negative	Corrected calcium	10.0 mg/dL
Sediment		Phosphate	4.0 mg/dL
Red blood cells	<1/HPF	Total bilirubin	0.4 mg/dL
White blood cells	5–9/HPF	Aspartate aminotransferase	15 U/L
Urinary biochemical tests		Alanine aminotransferase	13 U/L
Daily urinary protein	0.9 g/24 hr	Lactate dehydrogenase	192 U/L
N-Acetylglucosaminidase	21.1 IU/L	Alkaline phosphatase	136 U/L
*β*2-Microglobulin	36.07 mg/L	Creatin kinase	31 U/L
Complete blood count		Total cholesterol	224 mg/dL
White blood cells	7100/mL	LDL cholesterol	162 mg/dL
Neutrophils	65%	Triglyceride	110 mg/dL
Eosinophils	11%	Glucose	97 mg/dL
Basophils	0%	Hemoglobin A1c	6.0%
Lymphocytes	7%	Serology	
Monocytes	17%	C-reactive protein	1.71 mg/dL
Hemoglobin	11.2 g/dL	HBs antigen	Negative
Platelets	32.4 × 104/mL	Anti-HCV antibody	Negative
Coagulation tests		Immunoglobulin G	1723 mg/dL
PT-INR	1.06	Immunoglobulin A	194 mg/dL
APTT	30.4 sec	Immunoglobulin M	74 mg/dL
APTT, ctrl	32.5 sec	Immunoglobulin E	333 IU/mL
D dimer	1.4 *μ*g/mL	Anti-streptolysin O	21 IU/mL
Blood chemistry tests		Complement 3	139 mg/dL
Total protein	8.0 g/dL	Complement 4	52 mg/dL
Albumin	4.2 g/dL	CH50	64.0 U/mL
Urea acid	4.1 mg/dL	Rheumatoid factor	33 IU/mL
Urea nitrogen	23.9 mg/dL	Anti-nuclear antibody	×40>
Creatinine	2.01 mg/dL	Anti-dsDNA-Ab	<10 U/cmL

PT-INR, prothrombin time-international normalized ratio; APTT, activated partial thromboplastin time; LDL, low-density lipoprotein; HBs, hepatitis B surface; HCV, hepatitis C virus; CH50, 50% hemolytic unit of complement; DNA, deoxyribonucleic acid.

**Table 2:** Ten cases of biopsy proven acute tubulointerstitial nephritis from a single center in a rural area from 2008 to 2021.

Case #	Age	Sex	eGFR at diagnosis	Renal pathology	Uveitis	Initial treatment	Outcome
1	Teens	M	38.5	ATIN	No	Prednisolone 25 mg	eGFR 97.1 mL/min/1.73 m^2^

2	60 s	M	27.7	ATIN	Yes	Prednisolone 30 mg	Relapse+, eGFR 41.6 mL/min/1.73 m^2^

3	30 s	F	9.4	ATIN	Yes	Prednisolone 30 mg	eGFR 60.9 mL/min/1.73 m^2^

4	60 s	M	5.1	ATIN	Not examined	No steroid usage	eGFR 35.1 mL/min/1.73 m^2^

5	20 s	M	49.1	ATIN	Yes	Prednisolone 30 mg	eGFR 110.0 mL/min/1.73 m^2^

6	70 s	F	3.2	ATIN, Fibrosis	Not examined	Prednisolone 50 mg, HD	Relapse+, eGFR 23.1 mL/min/1.73 m^2^

7	20 s	M	31.9	ATIN	Yes	Prednisolone 20 mg	eGFR 68.1 mL/min/1.73 m^2^

8	Teens	F	43.7	ATIN, IgA deposition	Yes	Prednisolone 15 mg	eGFR 93.9 mL/min/1.73 m^2^

9	50 s	M	7.4	ATIN	Not examined	No steroid usage, HD	eGFR 46.3 mL/min/1.73 m^2^

10	40 s	F	11.1	ATIN	No	Prednisolone 30 mg	eGFR 20.8 mL/min/1.73 m^2^

ATIN, acute tubulointerstitial nephritis; HD, hemodialysis.

## Data Availability

The datasets generated during the current case series are available from the corresponding author on reasonable request.
